# Can the Future State of the Milk Be Predicted during Pregnancy

**Published:** 1842-11-19

**Authors:** 


*Can the future state of the Milk be predicted during Pregnancy?*—M. Donné is of opinion that it *can;* and in a very simple manner—by examining the state of the viscid, yellowish secretion which very generally, he says, may be squeezed out of the breasts of pregnant women, and to which the name of *colostrum* has been given. This imperfectly formed milk varies much both in quantity and in its physical properties in different cases; and M. Donné thinks that "he has ascertained that there is a very uniform relation between the nature of this fluid secreted during pregnancy, and the milk that is formed after delivery: or, in other words, that the examination of the colostrum and of its principal characters, enables us to predict what will be the condition of the mammary secretion, its quantity, and its essential properties."

The following are the chief conclusions to which his enquiries have led him:

1. In some women, scarcely a single drop of the *colostrum* can be made to exude from the mammæ during pregnancy. When this is the case, the secretion of the milk afterwards will "presqu' à coup sur," be scanty after delivery, and probably insufficient for the infant.

2. When the *colostrum* is abundant, but thin, watery, or like a thin mucilage, and does *not exhibit any striæ of a yellow-coloured, thick, and viscid matter,*—the italic letters are M. Donné's—the milk afterwards formed is almost always poor, and not nutritious.

3. When the colostrum is—in the eighth month of pregnancy, for example—moderately abundant, so that a few drops can be obtained from the mammæ without difficulty, and especially when it is found to contain distinct striæ* of a yellow-coloured, thickish matter, we may almost confidently predict that the milk will be sufficiently copious, and of good quality.—*Med. Chir Rev., from Conseils aux Meres, &c. Paris,* 1842.

*Remarks.*—We confess that we have our doubts whether experience will warrant the importance of the signs mentioned by M. Donné. He is one of those zealous, enthusiastic men, full of cleverness and hope, who are apt to arrive at positive conclusions rather more quickly than we slow-footed Englishmen quite approve of. But it is always well to know what our neighbours are thinking of and doing; as not a few useful hints may occasionally be derived from the very errors which they are apt to fall into.—*Rev.*

---

*Transfusion of the Blood of a Goat into the Veins of a Man.* By Dr. Blieding.—A man, 38 years of age, was seized with an hæmoptysis, which continued so long, and was so violent, that the only means of saving his life appeared to be by supplying the loss of blood by transfusion. On the fifth

* With the assistance of the microscope we often discover that the colostrum is rich in milky globules, already well formed, of a good size, without any admixture of mucous globules, and that it contains also a greater or less quantity of granular corpuscles.

